# The Effectiveness and Safety of Botulinum Neurotoxin in Obstetric Brachial Plexus Injury: A Systematic Review and Meta-Analysis

**DOI:** 10.3390/healthcare10122419

**Published:** 2022-11-30

**Authors:** Ting-Yen Chen, Yu-Chi Su, Yu-Ching Lin, Yao-Hong Guo

**Affiliations:** 1 National Cheng Kung University Hospital, College of Medicine, National Cheng Kung University, Tainan 70428, Taiwan; 2 Department of Physical Medicine and Rehabilitation, National Cheng Kung University Hospital, College of Medicine, National Cheng Kung University, Tainan 70428, Taiwan; 3 Department of Physical Medicine and Rehabilitation, College of Medicine, National Cheng Kung University, Tainan 70428, Taiwan

**Keywords:** botulinum neurotoxin, obstetric brachial plexus injury, meta-analysis

## Abstract

Obstetric brachial plexus injury, also known as neonatal brachial plexus injury, is not unusual in newborns. Given the lack of a comprehensive synthesis of the available data on the effectiveness of botulinum neurotoxin (BoNT) in treating children with obstetric brachial plexus injury, we conducted a systematic review and meta-analysis. We searched PubMed, Embase, Web of Science, and Cochrane databases from inception to 25 November 2022. Outcomes were function of the shoulder and elbow joints, muscle power of the deltoid, biceps brachii and triceps brachii, and the recurrence rate of subluxation or dislocation after reduction of the shoulder joint after BoNT application. Meta-regression was conducted to assess the moderator effect of age. We included 11 case series and 2 cohort studies. Passive range of motion of shoulder external rotation (standardized mean difference [SMD], 0.678; 95% confidence interval [95%CI], 0.423 to 0.934), Active Movement Scale for shoulder external rotation (SMD, 0.47; 95%CI, 0.131 to 0.808), and active range of motion of elbow extension (SMD, 2.445; 95%CI, 1.556 to 3.334) increased significantly after BoNT. However, the modified Gilbert scale for shoulder abduction (SMD, 1.239; 95% CI, −0.2 to 2.678), the Toronto score for active elbow flexion (SMD, 1.099; 95% CI, −0.053 to 2.252), muscle power of deltoid (SMD, 0.675; 95% CI, −0.599 to 1.949), biceps brachii (SMD, 0.225; 95% CI, −0.633 to 1.083), and triceps brachii (SMD, 1.354; 95% CI, −1.493 to 4.202) did not reach statistical significance. The moderator effect of age was not significant (*p* = 0.88). Meta-analysis was not done for recurrence rate of subluxation or dislocation due to insufficient data. In conclusion, our data support BoNT use in patients with obstetric brachial plexus injury. However, definite conclusions cannot be drawn due to small sample size and the lack of randomized controlled trials. More research is warranted to clarify the effectiveness of BoNT in patients with obstetric brachial plexus injury by using standardized injection protocols and outcome measurements.

## 1. Introduction

Obstetric brachial plexus injury (OBPI) is a type of peripheral neuropathy of the upper extremity that is typically caused by traction on the newborn during childbirth [1]. Due to its etiology, it is also known as brachial plexus birth injury or neonatal brachial plexus palsy [2,3]. Macrosomia, shoulder dystocia, and breech vaginal birth are the most common risk factors for OBPI. Its prevalence in developed countries is 0.5 to 2 per 1000 live births [4]. Although its incidence has been decreasing, and most patients with OBPI recover spontaneously, 10% to 30% of patients experience permanent muscle weakness or contracture [5,6,7]. The most common patterns of motor deficit in obstetric brachial plexus palsy with upper root involvement are shoulder abduction and external rotation because of paralysis of the deltoid, supraspinatus, infraspinatus, and teres minor muscles [8]. Some children may show inadequate elbow extension and even contracture [9]. More severe complications such as shoulder posterior subluxation and dislocation might also occur due to muscle imbalance [10]. Treatment of OBPI includes early rehabilitation in the first few months of life [11], and surgical treatment might be considered after 3 months of age based on the severity of function limitation [12]. Surgical intervention may include primary nerve surgery or secondary musculoskeletal surgery for situations caused by muscle imbalance or contracture [13]. Hence, it is reasonable to seek for a solution to decrease the imbalance between muscles to avoid the potential sequelae of OBPI, such as contracture of elbow, contracture, subluxation, and dislocation of the shoulder.

Botulinum neurotoxin (BoNT) blocks acetylcholine release at the presynaptic nerve terminals. By decreasing the muscle strength of the BoNT-injected muscles [14,15], the imbalance between muscles in patients with OBPI may be ameliorated. Theoretically, BoNT is a promising non-surgical treatment for OBPI to slow down disease progression and to increase patients’ quality of life. Several trials applying BoNT to subjects with OBPI have been conducted. In 2019, a systematic review concluded that BoNT can reduce internal rotation and adduction contractures of the shoulder, flexion and extension contractures of the elbow, and forearm pronation contracture, and these beneficial effects blunted when used in older patients [16]. However, the article did not perform a meta-analysis and made the conclusions by only narrative synthesis. We believe that it is necessary to carry out a formal meta-analysis to increase the validity of the evidence synthesis for the effectiveness of BoNT in OBPI [17]. Additionally, patient demographics such as age have been found to be correlated to BoNT effectiveness when applied for purposes other than OBPI [18,19], and whether similar relationships can be found in OBPI deserves exploration.

In this systematic review and meta-analysis, we investigated the effectiveness of BoNT for OBPI in children and identified potential moderators such as age at first injection. We believe that we could provide evidence for the use of BoNT in OBPI by revealing the improvement of shoulder function after BoNT injection in children with OBPI. We searched the Embase, PubMed, Web of Science, and Cochrane Library databases to include all available studies. We believe that we can find a significant improvement in the range of motion and function of shoulder and elbow joints as well as subluxation or dislocation of the shoulder joint after application of BoNT.

## 2. Materials and Methods

**Figure 1:**
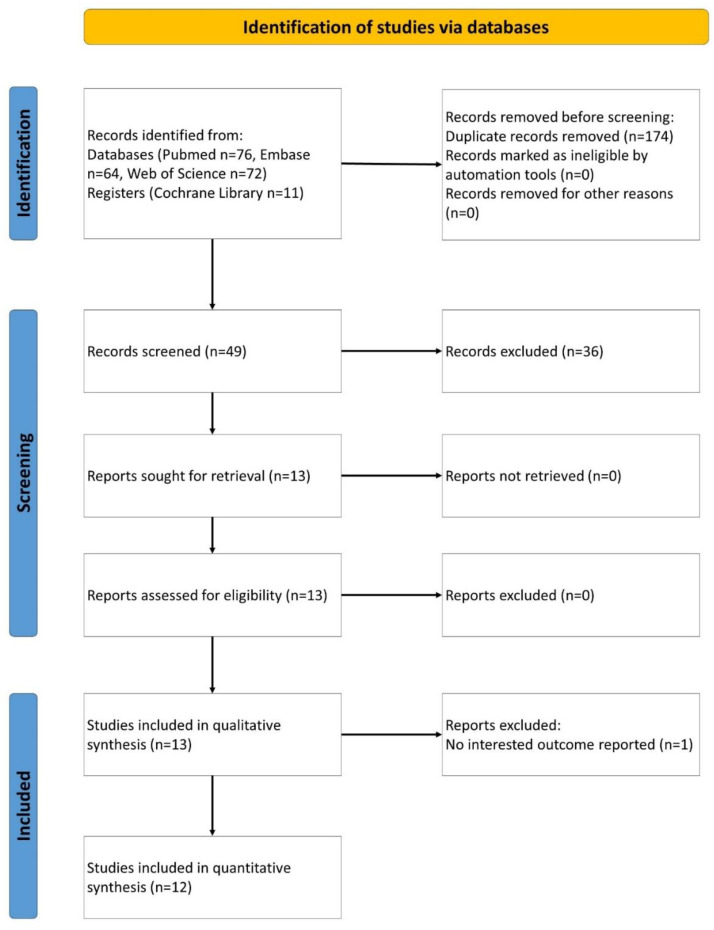
Literature screening process and results.

This systematic review and meta-analysis was conducted according to the Preferred Reporting Items for Systematic Review and Meta-Analysis (PRISMA) guidelines (See Table S1 for PRISMA checklist) [20]. The protocol was registered at the International Platform of Registered Systematic Review and Meta-Analysis Protocols (INPLASY). The registration number is INPLASY202290017. The process and results of literature screening are shown in Figure 1.

### 2.1. Eligibility Criteria

We included both randomized controlled trials and non-randomized controlled studies that recruited patients with OBPI with mean age under 18 years old. The case arm must include application of BoNT with or without combination of other interventions. No restrictions were set for the control arms. Outcomes should include ROM, function, subluxation or dislocation of shoulder or elbow joint. Only articles published in English were included. Studies not written in English and not reporting the etiology of brachial plexus injury were excluded.

### 2.2. Search Strategy

We searched the PubMed, Embase, Web of Science, and Cochrane Library databases from inception to 25 November 2022, using the key terms “brachial plexus injury” AND “botulinum toxin”. The detailed search terms were listed in the Table S2. The references of the selected articles were also manually searched for eligible studies.

### 2.3. Study Selection and Data Extraction

The first two authors (TYC and YCS) independently reviewed the titles and abstracts of applicable studies. If the authors had different opinions concerning the eligibility of studies, a consensus was reached through discussion. If an agreement could not be reached, the senior author (YCL) made the final decision. The following data were extracted from the selected studies: the first author, year of publication, demographic information, dosage of BoNT, site of injection, commercial form of BoNT, outcome measurement tool, pre-injection operation, adverse events, and time of last outcome measurement. We contacted the authors for further details if necessary.

### 2.4. Quality Assessment

We assessed the quality of the selected studies using the Joanna Briggs Institute Critical Appraisal Checklist for Case Series [21]. If disagreements occurred concerning risk identification, a consensus was reached through discussion. If a consensus could not be reached, the senior author (YHG) made the final decision. We used ReviewerManager version 5.3 (Cochrane, London, UK) to visualize the risk of bias as a graph.

### 2.5. Stastistical Analysis

The primary outcome was the improvement of function of the shoulder joint after BoNT injection. Secondary outcomes comprised the improvement of function of elbow joint, muscle power of the deltoid, biceps brachii and triceps brachii, and the recurrence rate of subluxation or dislocation after reduction of the shoulder joint with BoNT. The passive ROM, active ROM, modified Gilbert scale [22], Active Movement Scale (AMS) [23], Toronto score [24] of the interested joints, and the Medical Research Council (MRC) scale [25] of the target muscles before and after BoNT administration were compared and summarized with the random-effects model. Results were presented as standardized mean differences (SMDs) and 95% confidence intervals (CIs). The *I*^2^ value was used to assess between-study heterogeneity, and significant heterogeneity was identified when *I*^2^ value was above 50% [26]. Furthermore, we conducted a random-effects meta-regression to validate whether the primary outcome varied depending on the patient age at first injection. A sensitivity analysis was also performed for the primary outcome by removing one trial at a time and analyzing the remaining trials to determine whether the effect resulted from a single study. Finally, we used funnel plots and Egger’s test to assess publication bias [27], in which two-tailed *p* < 0.1 was regarded as statistically significant [28]. We used Comprehensive Meta-Analysis Software version 3 (Biostat, Englewood, NJ, USA) for all analyses.

### 2.6. Certainty of Evidence

We used the Grading of Recommendations Assessment, Development, and Evaluation (GRADE) to assess the certainty of the evidence of our primary outcome. The final rating depended on the overall risk of bias, imprecision, inconsistency, indirectness, and publication bias [29].

## 3. Results

### 3.1. Study Selection and Description

We identified 49 articles in the initial search, of which 13 met our inclusion criteria (Figure 1), including 11 case series [10,30,31,32,33,34,35,36,37,38,39] and 2 cohort studies [40,41]. Overall, 347 patients with OBPI were treated with BoNT. The information of these studies is summarized in Table 1.

### 3.2. Risk of Bias Assessment

**Figure 2:**
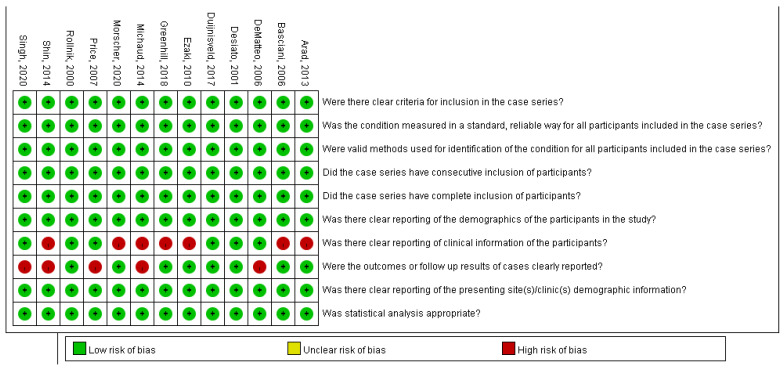
Summary graph of risk of bias assessment [10,30,31,32,33,34,35,36,37,38,39,40,41].

Out of the 13 included studies, 2 studies did not report their participants’ operation history before BoNT injection [32,35] and 6 studies did not report average dose of BoNT injection [10,34,35,36,37,39]. The operation history means any sort of surgery intended to relieve the symptoms of OBPI done before the trials. Hence, they scored high risk of bias for not clearly reporting the clinical information of participants. Four articles did not clarify the last time of outcome measurement after BoNT injection [36,37,39,40]. The four articles had different last follow-up time points for each individuals, and they only reported the mean of the last follow-up time. These articles did not analyze the potential reasons and influence of the difference of follow-up time lengths between individuals, so we believe that this may cause a high risk of reporting bias. Hence, they scored high risk of bias for not clearly reporting the follow-up results of cases. Further details were presented in Figure 2.

### 3.3. Outcomes

#### 3.3.1. Shoulder Joint

**Figure 3:**
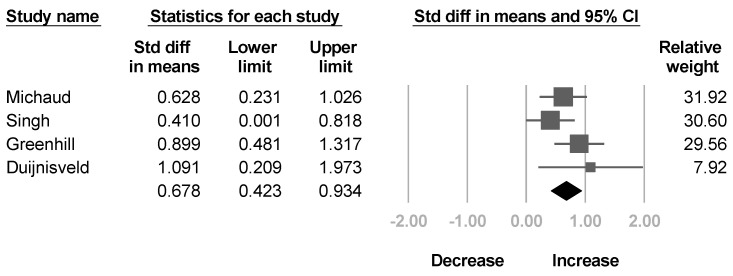
Forest plot of change of passive range of motion of shoulder external rotation after botulinum neurotoxin.

Four studies [36,37,38,41] reported the passive ROM of shoulder external rotation before and after BoNT injection. The summarized effect size showed a significant increase in passive ROM of shoulder external rotation without significant heterogeneity (SMD, 0.678; 95% CI, 0.423 to 0.934, *I*^2^ = 17.4%; Figure 3).

**Figure 4:**
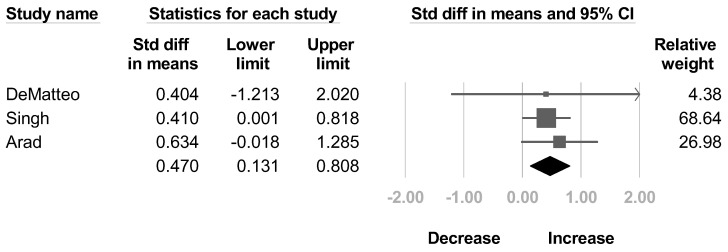
Forest plot of change of Active Movement Scale of shoulder external rotation after botulinum neurotoxin.

Three articles [33,34,38] reported the AMS for shoulder external rotation. The results revealed a significant improvement in AMS score after BoNT without significant heterogeneity (SMD, 0.47; 95% CI, 0.131 to 0.808, *I*^2^ = 0.000%; Figure 4).

**Figure 5:**
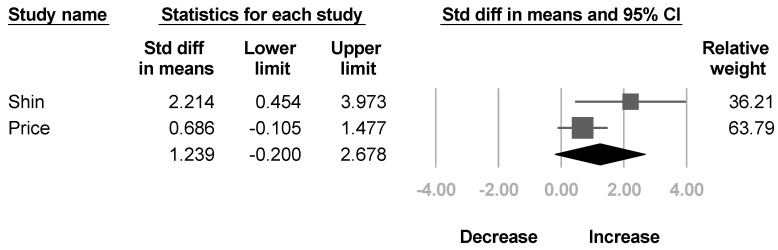
Forest plot of change of modified Gilbert scale after botulinum neurotoxin.

Two papers [35,40] reported the modified Gilbert scale for shoulder abduction. The pooled estimate did not reach statistical significance, and the heterogeneity between studies were also significant (SMD, 1.239; 95% CI, −0.2 to 2.678, *I*^2^ = 58.5%; Figure 5).

#### 3.3.2. Elbow Joint

**Figure 6:**
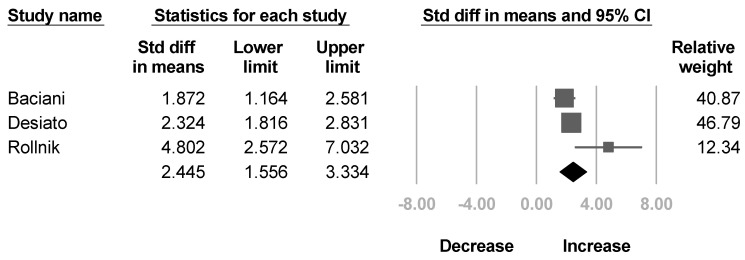
Forest plot of change of active range of motion of elbow extension after botulinum neurotoxin.

Three trials [30,31,32] mentioned the active ROM of elbow extension. The results revealed a significant increase in active ROM of elbow extension with significant heterogeneity (SMD, 2.445; 95% CI, 1.556 to 3.334, *I*^2^ = 67.8%; Figure 6).

**Figure 7:**
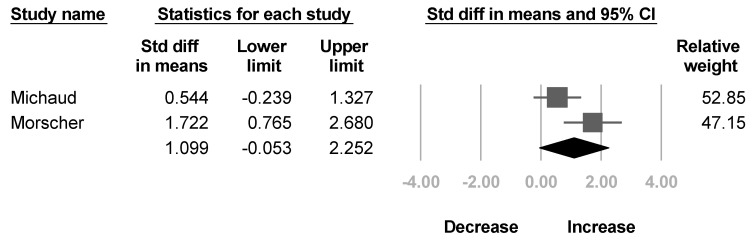
Forest plot of change of Toronto score for elbow flexion after botulinum neurotoxin.

Two studies [36,39] reported the Toronto score for active elbow flexion. No significant improvement in active elbow flexion was found after BoNT, and the heterogeneity reached statistical significance (SMD, 1.099; 95% CI, −0.053 to 2.252, *I*^2^ = 71.3%; Figure 7).

#### 3.3.3. Muscle Strength

**Figure 8:**
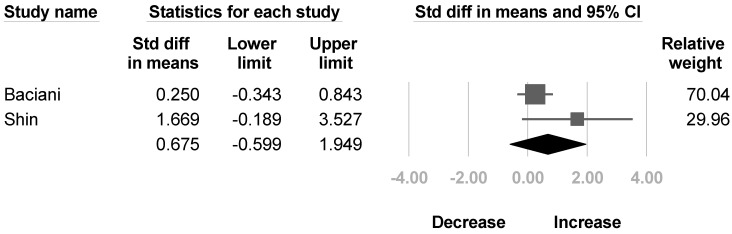
Forest plot of change of muscle strength of deltoid after botulinum neurotoxin.

**Figure 9:**
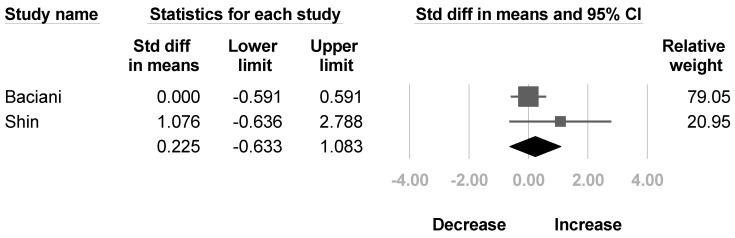
Forest plot of change of muscle strength of biceps brachii after botulinum neurotoxin.

**Figure 10:**
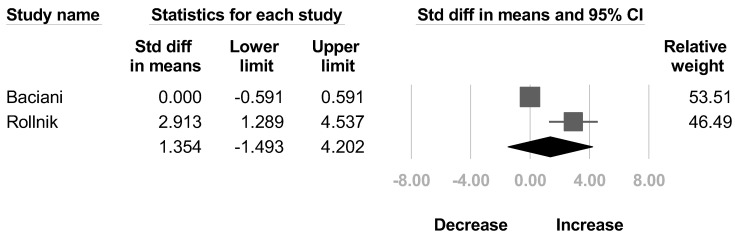
Forest plot of change of muscle strength of triceps brachii after botulinum neurotoxin.

Two articles [32,35] mentioned the change of muscle strength of the deltoid and biceps brachii after BoNT injection. Another two studies [30,32] reported the muscle strength of triceps brachii. No significant difference were found in the deltoid (SMD, 0.675; 95% CI, −0.599 to 1.949, *I*^2^ = 50.8%; Figure 8), biceps brachii (SMD, 0.225; 95% CI, −0.633 to 1.083, *I*^2^ = 26.2%; Figure 9), and triceps brachii (SMD, 1.354; 95% CI, −1.493 to 4.202, *I*^2^ = 90.8%; Figure 10) after BoNT.

#### 3.3.4. Subluxation or Dislocation after Reduction of the Shoulder Joint

One paper [10] concluded that BoNT was a useful adjunct to the treatment of early posterior subluxation or dislocation of the shoulder joint in infants with OBPI. Meta-analysis was not conducted due to insufficient data.

#### 3.3.5. Meta-Regression, Sensitivity Analysis and Publication BIAS Assessment

**Figure 11:**
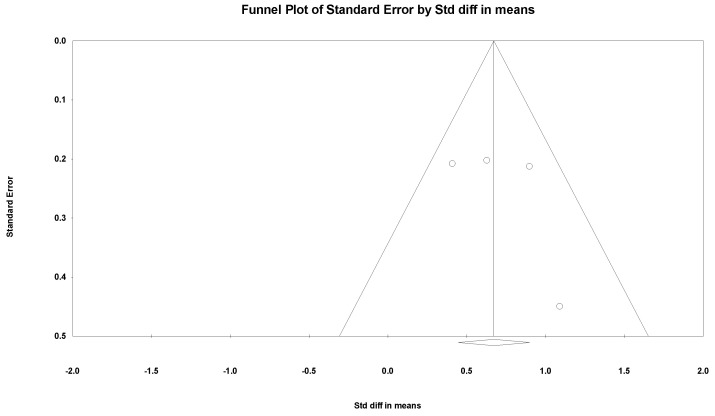
Funnel plot of change of passive range of motion of shoulder external rotation after botulinum neurotoxin. Dots indicate studies, and the diamond indicates the summarized effect size.

The meta-regression, sensitivity analysis, and publication bias were only conducted for passive ROM of shoulder external rotation due to insufficient data of the other outcomes. The meta-regression analysis did not reveal a significant relationship between age and the improvement of passive ROM of shoulder external rotation (*p* = 0.88). In the sensitivity analysis, the lowest SMD was 0.139 (95% CI, 0.304 to 0.849) when the study by Greenhill et al. [37] was excluded, and the highest was 0.203 (95% CI, 0.423 to 0.934) when the study by Michaud et al. [36] was excluded. No publication bias was detected by the funnel plot (Figure 11) and Egger’s test (*p* = 0.47).

#### 3.3.6. Certainty of Evidence

The certainty of evidence of an increase in passive ROM of shoulder external rotation resulting from BoNT was rated low. The rating started from moderate because all of the included studies were not randomized controlled trials. The rating was further downgraded due to high risk of bias (Table 2).

## 4. Discussion

In our systematic review and meta-analysis, BoNT increased the active and passive range of motion of external rotation of the shoulder and the range of active extension of the elbow. The changes of range of active shoulder abduction, range of active elbow flexion, and muscle strength of deltoid, biceps brachii and triceps brachii did not reach statistical significance after BoNT. The treatment effect of BoNT in external rotation of the shoulder was not related to the age of participants.

Some of the outcomes in our meta-analysis did not reach statistical significance. We believe that these may result from type II error due to insufficient data. All of these outcomes had a trend of improvement after BoNT injection, though the large confidence intervals crossed zero, making them non-significant. More studies are warranted to fill this knowledge gap.

Unlike the previous systematic review published in 2019 [16], we did not find a relationship between age and the effectiveness of BoNT in OBPI. The systematic review made their conclusion based on a single case series in 2006 by Basciani et al., which included 22 children [32]. Basciani et al. made their conclusion by the finding that four older children responded poorly to BoNT. Although we included much more participants, we still could not reproduce their findings. Larger trials with rigorous designs are necessary to find out the effectiveness of BoNT for OBPI in older children.

Aside from improving the movement of the upper limbs, BoNT in OBPI may also have the potential to help avoid the need of surgical intervention for subluxation, dislocation, and contracture. It is not uncommon for patients with OBPI to undergo musculoskeletal surgery for situations caused by muscle imbalance or contracture [13]. Hence, it is reasonable to focus more on the role of BoNT in avoiding surgery. However, there was only one article [10] published so far which reported the subsequent rate of surgical intervention after BoNT. We encourage future research to focus more on this aspect of BoNT in OBPI.

No severe adverse events were reported in the included studies. The most common side effects were transient weakness and pain. This may reassure future research to apply BoNT in these relatively young patients. However, the low number of participants in the individual research may mask potential serious adverse events. Moreover, five [33,35,36,38,40] articles did not report the details of adverse events. Future trials should still be careful when applying BoNT in patients with OBPI.

Our article of meta-analysis has several strengths. First, this was the first meta-analysis to analyze the effectiveness and safety of BoNT for treating OBPI. Second, ours is the first study to provide results of meta-regression between BoNT effectiveness and patient age at first injection.

This study has several limitations. First, the statistical power was limited by the small sample size and low evidence level of the included articles. Second, the scenario and timing of BoNT injections differed within and between studies, making it difficult to carry out subgroup analysis to compare the effectiveness of BoNT in different clinical scenarios. Third, the injection protocols differed in terms of dosage, injection site, and commercial forms of BoNT. Some articles calculated their dose of BoNT per muscle, while other studies only reported the total dose of individual subject. Hence, we could not propose an optimal protocol for BoNT injection according to the existing evidence. Moreover, the absence of accurate reporting of drug doses may also impact our results. Fourth, all of the papers included in our meta-analysis were non-randomized controlled studies, which impeded the grade of certainty of our results. Fifth, most available studies combined BoNT with conventional rehabilitation, and the contents of these therapies varied between trials. Whether treatments other than BoNT affected the effectiveness of BoNT remained unknown. Finally, ten different outcome measurement tools were used across the 13 included articles, further attenuating the statistical power of our study. Future research should consider to use similar outcome measurements to increase reproducibility and generalizability.

## 5. Conclusions

In conclusion, our meta-analysis recommended the use of botulinum neurotoxin in patients with obstetric brachial plexus injury. However, definite conclusions cannot be drawn due to small sample sizes and the lack of randomized controlled trials. Currently, there is still a significant gap in the literature in this area. Although our study may partially fill such knowledge gap, future studies with homogenous participants, clearly defined injection indications, and standardized injection protocols, such as the dose of BoNT and dilution methods, are still warranted to verify the effectiveness and safety of BoNT in OBPI.

## Figures and Tables

**Table 1:** Details of included articles.

Research (First Author, Year of Publication)	Country	Study Design (Number of Participants)	Mean Age at the Time of First Injection (Years Old)	Gender (Percentage of Male)	Mean Dosage of BoNT	Site(s) of Injection	Commercial Forms of BoNT (Company)	Outcome Measurements	Pre-Injection Operation (n, Percentage)	Percentage of Adverse Event	Last Time of Outcome Measurement (Months Post-Injection)
Rollnik, 2000 [30]	Germany	Case series (6)	3.17	0	39.2 units/muscle	Triceps	Dysport (Ipsen)	Triceps MRC, active ROM of elbow extension	nerve repair (3, 50%)	0	12
Desiato, 2001 [31]	Italy	Case series (50)	4.7	52	14.4 units/kg	Pectoralis major, pectoralis minor, teres major, Biceps brachii, Brachialis, Pronator teres, Subscapularis, latissimus dorsi, brachioradialis	Dysport (Ipsen)	Active ROM of elbow extension, shoulder external rotation, Global Clinical Rating Scale	tendon lengthening (5, 10%)	2 (transient weakness)	9
Basciani, 2006 [32]	Italy	Case series (22)	5.6	54.5	22 units/kg	Pectoralis major, Biceps brachii, Brachialis, Pronator teres	Dysport (Ipsen)	MRC of biceps, triceps and deltoid active ROM of elbow extension, Mallet score, Nine-Hole Peg Test (NHPT)	N/R	9.1 (articular pain)	12
DeMatteo, 2006 [33]	Canada	Case series (8)	1.04	62.5	4 units/kg/muscle	Triceps, pectoralis major, latissimus dorsi	Botox (Allergan)	AMS of elbow shoulder flexion, abduction and total external rotation	tendon lengthening (4), nerve repair (3)	N/R	4
Price, 2007 [40]	USA	Retrospective cohort (13 in treatment group)	5.8 (treatment group)	46.2 (treatment group)	100 units/muscle	pectoralis major	N/A	Modified Gilbert score for shoulder abduction	tendon transfer (13, 100%)	N/R	N/R (Mean follow-up time 36)
Ezaki, 2010 [10]	USA	Case series (35)	0.48	48.6	N/R (2–3 units/kg/muscle)	latissimus dorsi, pectoralis major, subscapularis, teres major	Botox (Allergan)	Recurrence rate after reduction for subluxation and dislocation	None (0, 0%)	0	12
Arad, 2013 [34]	Canada	Case series (27)(19 shoulder, 8 elbow)	2.69	29.6	N/R (40–50 units/muscle for shoulder; 60–75 units/muscle for elbow)	latissimus dorsi, pectoralis major, subscapularis, triceps	Botox (Allergan)	AMS of elbow flexion, shoulder external rotation	nerve repair (17, 63%)	0	12
Shin, 2014 [35]	Korea	Case series (4)	6.65	N/R	N/R (2–3 units/kg/muscle)	triceps brachii, pectoralis major	Botox (Allergan)	MRC of biceps and deltoid, Modified Gilbert score	N/R	N/R	1
Michaud, 2014 [36]	USA	Case series (59)	3.02	52.5	N/R (up to 10 units/kg)	latissimus dorsi, pectoralis major, subscapularis, triceps, biceps, pronator teres, flexor carpi ulnaris	Botox (Allergan)	Passive ROM of shoulder external rotation, Toronto score for elbow flexion and extension, Mallet score	nerve repair (28), tendon release (8), tendon transfer (4)	N/R	N/R (Mean follow-up time 13.2)
Duijnisveld, 2017 [41]	Netherland	Prospective cohort (15 in treatment group)	2.53 (treatment group)	66.7 (treatment group)	2 units/kg	subscapularis	Botox (Allergan)	Passive ROM of shoulder external rotation	nerve repair (10, 75%)	0	3
Greenhill, 2018 [37]	USA	Case series (49)	0.96	N/R	N/R (up to 10 units/kg)	latissimus dorsi, pectoralis major, subscapularis	Botox (Allergan)	Passive ROM of shoulder external rotation	None (0, 0%)	0	N/R (Mean follow-up time 21.1)
Singh, 2020 [38]	USA	Case series (47)	1	44.7	7.4 units/kg	latissimus dorsi, pectoralis major, subscapularis	OnabotulinumtoxinA (Allergan)	AMS of shoulder external rotation, Passive ROM of shoulder external rotation	nerve repair (20, 42.6%)	N/R	11
Morscher, 2020 [39]	USA	Case series (12)	0.33	N/R	N/R (range: 10–30 units/muscle)	triceps	OnabotulinumtoxinA (Allergan)	Toronto score elbow flexoin	None (0, 0%)	0	N/R (Mean follow-up time 72)

AMS: Active Movement Scale, BoNT: botulinum neurotoxin, MRC: Medical Research Council scale, ROM: range of motion, N/A: not applicable, N/R: not reported.

**Table 2:** Certainty of evidence for passive range of motion of shoulder external rotation.

Quality Assessment						Summary of Findings, SMD (95% CI)	
Number of Participants (Studies), Follow-Up Period	Risk of Bias	Inconsistency	Indirectness	Imprecision	Publication Bias	Passive ROM of Shoulder External Rotation	Certainty of Evidence
170 (4), till loss of follow or surgical intervention	Serious limitation ^a^	No serious limitation ^b^	No serious limitation ^c^	No serious limitation ^d^	Undetectable	0.672 (0.444, 0.899)	Low ⨁⨁◯◯ ^e^

BoNT, botulinum neurotoxin; CI, confidence interval; SMD, standardized mean difference. ^a^ Half of the included studies scored high risk of bias in at least one category. ^b^ I^2^ score was below 50%. ^c^ No indirectness was detected in this outcome. ^d^ The upper and lower limit of 95% CI both favored BoNT. ^e^ ⨁◯◯◯ indicates very low, ⨁⨁◯◯ indicates low, ⨁⨁⨁◯ indicates moderate, and ⨁⨁⨁⨁ indicates high certainty of evidence.

## Data Availability

No new data were created in this study.

## Supplementary Materials

The following supporting information can be downloaded at: https://www.mdpi.com/article/10.3390/healthcare10122419/s1. Table S1: PRISMA checklist and search strategies for systematic review and meta-analysis. Table S2: Search terms for systematic review and meta-analysis. Ref [20] was cited in the Supplementary Materials.
